# Zfp580 Regulates Paracrine and Endocrine Igf1 and Igfbp3 Differently in the Brain and Blood After a Murine Stroke

**DOI:** 10.3389/fphys.2022.887180

**Published:** 2022-04-26

**Authors:** Christian J. Hoffmann, Melanie T. C. Kuffner, Janet Lips, Stephanie Lorenz, Matthias Endres, Christoph Harms

**Affiliations:** ^1^ Klinik und Hochschulambulanz Für Neurologie Mit Experimenteller Neurologie, Charité-Universitätsmedizin Berlin, Corporate Member of Freie Universität Berlin, Humboldt-Universität Zu Berlin, Berlin Institute of Health, Berlin, Germany; ^2^ Center for Stroke Research Berlin, Charité-Universitätsmedizin Berlin, Berlin, Germany; ^3^ German Center for Cardiovascular Research (DZHK), Partner Site Berlin, Berlin, Germany; ^4^ NeuroCure Clinical Research Center, Charité-Universitätsmedizin Berlin, Berlin, Germany; ^5^ German Center for Neurodegenerative Diseases (DZNE), Berlin, Germany; ^6^ Einstein Center for Neuroscience, Berlin, Germany

**Keywords:** ZFP580, IGF1, IGFBP3, stroke, paracrine, endocrine

## Abstract

Insulin-like growth factor 1 (Igf1) and insulin-like growth factor binding protein 3 (Igfbp3) are endocrine and paracrine factors that influence stroke occurrence, severity, and recovery. Low levels of endocrine Igf1 and Igfbp3 were associated with larger infarct volumes and unfavorable outcomes. Paracrine Igf1 is brain cytoprotective and improves functional recovery after stroke. In this study, we evaluated the effects of zinc finger protein 580 (Zfp580) on endocrine and paracrine Igf1 and Igfbp3 after stroke. Zfp580 suppressed the expression of Igf1 and Igfbp3 in cerebral microvascular endothelial cells (bEnd.3) as determined by real-time RT-PCR. Zfp580 was suppressed by combined oxygen and glucose deprivation (OGD) and mediated the effect of OGD on Igf1 and Igfbp3. *In vivo*, we evaluated paracrine regulation by real-time RT-PCR of brain lysates and endocrine regulation by ELISA of blood samples. Genomic ablation of Zfp580 did not alter basal paracrine or endocrine Igf1 and Igfbp3 levels. After transient middle cerebral artery occlusion (MCAo), Zfp580 was globally elevated in the brain for up to 3 days. Paracrine Igf1 and Igfbp3 were selectively induced in the ischemic hemisphere from day 2 to day 3 or day 1 to day 7, respectively. In Zfp580 knockout mice, the paracrine regulations of Igf1 and Igfbp3 were attenuated while endocrine Igf1 and the molar Igf1/Igfbp3 ratio were increased. In conclusion, Zfp580 differentially controls paracrine and endocrine Igf1 and Igfbp3 after stroke. Inhibition of Zfp580 might be a new treatment target leading to increased activity of Igf1 to improve stroke outcome.

## Introduction

Insulin-like growth factor 1 (Igf1) and insulin-like growth factor binding protein 3 (Igfbp3) affect stroke occurrence and severity, as well as stroke recovery. Igf1 has both endocrine and paracrine effects on the brain (Fernandez and Torres-Aleman, 2012; Gubbi et al., 2018). Endocrine circulating Igf1 can cross the blood-brain barrier at the choroid plexus (Carro et al., 2005). However, the majority of Igf1 within the brain is produced by neurons, glia, and vessels and secreted in a paracrine manner (Bondy et al., 1992; Lobie et al., 1993; Ashpole et al., 2015). In the bloodstream, Igf1 is bound to Igf-binding proteins (Igfbp), mainly to Igfbp3, resulting in the inactivation of Igf1 (Rajaram et al., 1997; Baxter, 2000). The liver is the primary source of Igfbp3 (Arany et al., 1994). Furthermore, endothelial cells express Igfbp3 in homeostasis. Its expression is elevated in cerebral microvascular endothelial cells following traumatic or hypoxic injury, but decreased in neurons after hypoxia (Walter et al., 1997; Lee et al., 1999). Igfbp3 inhibits the proliferation of neural progenitor cells (Kalluri and Dempsey, 2011). It also stops cell growth and causes apoptosis, either by blocking Igf1 or Igf1-independently by Igfbp3-specific cell surface proteins or receptors (Grimberg and Cohen, 2000).

In humans, growing evidence shows effects of endocrine Igf1 and Igfbp3 after stroke. However, there are no studies about specific effects of post-ischemic paracrine cerebral Igf1 or Igfbp3. It has been shown that low circulating Igf1 levels were associated with higher stroke incidence (Saber et al., 2017). Other studies, however, found no correlation between Igf1 and stroke incidence, although low Igfbp3 levels, which enhance free circulating Igf1, were linked to a higher risk of stroke (Johnsen et al., 2005; Kaplan et al., 2007). Concerning stroke severity, several studies have shown that circulating Igf1 and Igfbp3 were reduced after stroke and that post-stroke Igf1 and Igfbp3 serum levels were inversely associated with infarct volume (Schwab et al., 1997; Mackay et al., 2003; Denti et al., 2004; Tang et al., 2014). Low circulating Igf1 and Igfbp3 levels were predictive for unfavorable outcome (Silva-Couto Mde et al., 2014; Tang et al., 2014; Ebinger et al., 2015) and high levels of circulating Igf1 were predictive for better outcome (Bondanelli et al., 2006; Aberg et al., 2011; De Smedt et al., 2011). However, Ambrust et al. showed the opposite: high circulating Igf1 levels after stroke were predictive for an unfavorable outcome (Armbrust et al., 2017).

The paracrine effects of Igf1 were studied in rodent models: intracerebroventricular administration of Igf1 led to improved functional outcome and reduced neuronal loss (Guan et al., 1993; Schabitz et al., 2001; Mackay et al., 2003). Intracerebroventricular injection of Igf1 coding adeno-associated viral vectors improved neurogenesis and functional recovery (Zhu et al., 2008; Liu et al., 2017). Proliferating microglia (Ploughman et al., 2005; Lalancette-Hebert et al., 2007) and astrocytes express Igf1 after stroke (Yan et al., 2006). Moreover, systemic Igf1 has additional protective effects on cerebro-microvascular functions: selective reduction of systemic Igf1 by post-developmental liver specific knockdown of Igf1 impaired neurovascular coupling (Toth et al., 2015). In contrast, higher systemic Igf1 levels prior stroke were correlated to larger infarct size in mice (Endres et al., 2007). In summary, the effects of Igf1 and Igfbp3 on stroke outcome may be affected by their temporal and spatial regulation.

Zincfinger protein 580 (Zfp580) is a transcription factor that is involved in inflammation, angiogenesis and signaling triggered by hypoxia (Sun et al., 2010; Hoffmann et al., 2011; Meng et al., 2014; Stenzel et al., 2018). As recently demonstrated, Zfp580 may play a role in Igfbp3 regulation (Yin et al., 2022). In this study, we examined the effect of Zfp580 on post-ischemic paracrine and endocrine Igf1/Igfbp3 signaling.

## Methods

### Cell Culture

Brain microvascular endothelial cells (bEnd.3, ATCC CRL-2299) were purchased from ATCC and cultured in Dulbecco’s Modified Eagle’s Medium (DMEM, ATCC) supplemented with 10% FCS, L-glutamine and antibiotics. Cell clones with increased Zfp580 expression or expression of an shRNA embedded miRNA targeting Zfp580 or LacZ were generated using lentiviral particles. For a detailed description of lentiviral particle production, see Dätwyler et al. (Datwyler et al., 2011). Transfer vectors additionally encoded enhanced green fluorescent protein (EGFP) and puromycin resistance proteins separated by T2A self-cleavage peptides. After transduction, the cells were cultivated in DMEM with puromycin until 100% of the cells expressed EGFP. For experiments, the cells were cultured in supplemented DMEM without puromycin.

Lentiviral transfer vectors were designed based on a modified flap-Ubiquitin promoter-driven EGFP-WRE lentiviral transfer plasmid (pFUGW) [the FUGW plasmid was a gift from David Baltimore (Addgene plasmid #14883; http://n2t.net/addgene:14883; RRID:Addgene_14883)] (Lois et al., 2002). Zfp580, derived as a product of gene synthesis (Eurofins, Ebersberg, Germany), was followed by a T2A self-cleaving peptide sequence that was inserted by unidirectional cloning such that it preceded the sequences for EGFP and puromycin resistance in pFUGW. Small hairpin RNA (shRNA)-embedded microRNA for the expression of LacZ or Zfp580 targeting shRNA were designed using BLOCK-iT RNAi Designer (Invitrogen, USA) with the accession number NM_026900, which encodes murine Zfp580, and blasted against *Mus musculus* to control off-target effects. Annealing and ligation of shRNA targeting Zfp580 at nucleotide position 930 in the 5′ untranslated region (UTR) (Eurofins, Ebersberg, Germany) were performed using sticky ends in a BbsI-predigested vector (pcDNA6.2-GW/EmGFP with spectinomycin as a selection marker) following the manufacturer’s instructions (BLOCK-iT Pol II miR RNAi expression vector kit, Invitrogen). The ligated shRNAs were subcloned *via* BlpI and XhoI (204 bp-long fragments) into a modified version of the pFUGW lentiviral transfer vector for equal coexpression of the shRNA and EGFP (reporter protein) alongside puromycin. A LacZ nontargeting shRNA served as a control.

All transfer vectors and detailed steps for cloning and sequence verification are available upon request from the corresponding authors or *via* Addgene.

### Real-Time RT-PCR

RNA was extracted using TRIzol (Invitrogen) and stored at -80 °C prior to transcription. One microgram of RNA was transcribed with MLV reverse transcriptase (Promega) using random primers (Roche) and oligo-dT primers (Eurofins-MWG). Real-time PCR was performed with exon-spanning primers for tyrosine 3-monooxygenase/tryptophan 5-monooxygenase activation protein (Ywhaz) and intron-spanning primers for succinate dehydrogenase complex, subunit A, flavoprotein (Sdha) as internal controls (Gubern et al., 2009), and intron-spanning primers for Zfp580, Igf1 or Igfp3 using SYBR Green (Qiagen). For primer sequences, see Table 1. Comparable primer efficiency was confirmed. Melting curves were analyzed for all runs. Experiments in the absence of a template and those with untranscribed RNA served as negative controls. The ddCT method was used to figure out how many fold differences there were in mRNA levels compared to internal control levels.

**TABLE 1:** Primer sequences.

Gene Name	Forward primer	Reverse primer
Zincfinger protein 580 intron-spanning	ccc​cgg​acc​cga​gag​gct​g	gag​agg​agg​acc​gag​ggt​ggg
Insulin-like growth factor 1 intron-spanning	gtt​cgt​gtg​tgg​acc​gag​ggg	aca​atg​cct​gtc​tga​ggt​gcc​c
Insulin-like growth factor binding protein 3 intron-spanning	cct​acc​tcc​cct​ccc​aac​ctg​c	ggt​cac​tcg​gtg​tgt​gct​ggg
succinate dehydrogenase complex, subunit A, flavoprotein intron-spanning	ggg​gag​ccc​atg​cca​ggg​aa	cct​cca​gtg​ttc​ccc​aaa​cgg​c
tyrosine 3-monooxygenase/tryptophan 5-monooxygenase activation protein exon-spanning	cct​cgc​gct​ttt​ccc​agc​ctt	gca​cga​tga​cgt​caa​acg​ctt​ct

### ELISA

ELISAs to determine Igf1 and Igfbp3 (R&D Systems) levels in mouse blood serum were performed according to the manufacturer’s instructions. The Igf1: Igfbp3 molar ratio was calculated as previously described (Soubry et al., 2012).

### Oxygen and Glucose Deprivation

Endothelial cells were subjected to combined OGD as described previously (Harms et al., 2007). In brief, cultures were placed in an “*IN VIVO*_2_ 300” OGD chamber (Ruskinn, Pencoed, United Kingdom) with 5% CO_2_/0.3% O_2_ in buffer free of glucose for 4 h. Control cells were cultured for 4 h under normoxic conditions in the same buffer but supplemented with 5.5 mM glucose.

### Transient Middle Cerebral Artery Occlusion

All experimental procedures were approved by the local authorities (Landesamt für Gesundheit und Soziales, LaGeSo, Berlin (Reg G0254/16)) and were conducted following the German animal protection law and local animal welfare guidelines. Zfp580tm1a^(EUCOMM)Wtsi^ mice (constitutive Zfp580-knockout mice, C57/BL6/N background) were purchased from EuMMCR (Helmholtz Zentrum München). Transient filamentous blockage of the middle cerebral artery was performed on mice for 30, 45 or 60 min using a documented standard operating procedure from our laboratory (Endres et al., 2003). In brief, mice were anaesthetized with 1.5–3.5% Isoflurane and maintained in 1.0–2.5% Isoflurane with approximately 75/25 N_2_O/O_2_ and for pain relief, bupivacain gel (1%) was topically applied to the wound. The common carotid artery (CCA) external carotid artery (ECA) were ligated. A 180 µm diameter Doccol^®^ filament was introduced into the CCA and advanced up the internal carotid artery (ICA) to occlude the middle cerebral artery (MCA) for the indicated durations. For sham procedure, the filament was inserted to occlude the MCA and withdrawn immediately. Successful induction of stroke was proven by MRI-scans 24 h after surgery. For time course experiments, we used 3 male C57BL6/N wild-type mice for sham surgery, 4 mice for 30 min MCAo and 5 mice for 60 min MCAO for each reperfusion time as indicated in the respective figure (Figure 4). According to preset exclusion criteria concerning humane endpoints, 1 animal was excluded in the group of 7 days of reperfusion after 30 min MCAo and 1 animal in the group of 48 h as well as 2 animals in the group of 72 h of reperfusion after 60 min MCAO. For 45 min MCAo experiments with Zfp580tm1a and wild-type littermates, we used 6 age- and gender-matched animals in each group. None of these animals had to be excluded.

### Methods to Prevent Bias

Animals were randomized for MCAo operation, and experimenters were blinded to the genotype. Reporting conforms to the ARRIVE guidelines.

### Statistical Analyses

All data are presented as scatter dot plots with the mean ± standard deviation. The data were analyzed with GraphPad Prism version 8.2.0 and tested for normal distribution using the Shapiro-Wilk test. A detailed description of the corresponding statistical analysis is provided in the figure legends.

## Results

### Zfp580 Suppresses Igf1 and Igfbp3 in Cerebral Microvascular Endothelial Cells

**FIGURE 1:**
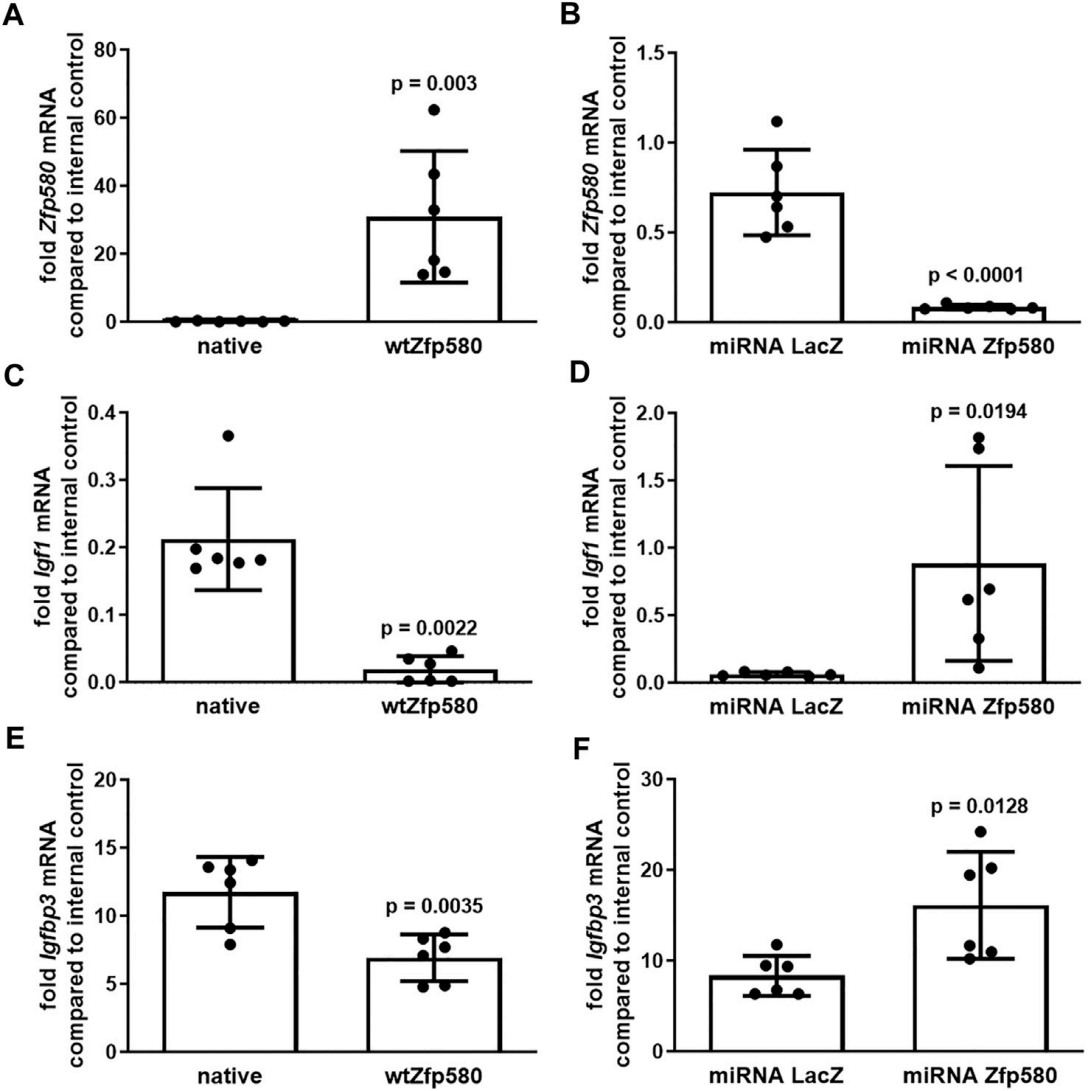
Zfp580 suppresses Igf1 and Igfbp3 in endothelial cells. Real time RT-PCR of native bEnd.3 or bEnd.3 with increased expression of *Zfp580* **(A,C,E)** or bEnd.3 expressing LacZ specific miRNA embedded shRNA as a negative control or expressing Zfp580 specific shRNA for knock down of *Zfp580* **(B,D,F)**. Increased expression of *Zfp580* leads to suppression of *Igf1* **(C)** and *Igfbp3* **(E)**. Knockdown of *Zfp580* leads to induction of *Igf1* **(D)** and *Igfbp3* **(F)**. The graphs show the means and scatter dot plots of independent experiments with standard deviations, as well as the two-tailed unpaired Students t-test **(A,B,D–F)** or Mann-Whitney test **(C)**.

We used the cerebral microvascular endothelial cell line bEnd.3. By lentiviral transduction, we generated cell clones with increased expression of Zfp580 or cell clones expressing a Zfp580 specific shRNA embedded in a miRNA cassette. A cell clone expressing miRNA embedded shRNA against LacZ served as negative control for knock down experiments. We determined the mRNA expression levels using real time RT-PCR. We achieved a strong increase of Zfp580 expression (Figure 1A) and knocked down Zfp580 efficiently by approximately 85% using RNAi (Figure 1B). Increased expression of Zfp580 led to a strong suppression of *Igf1* and *Igfbp3* mRNA (Figures 1C,E), whereas knock down of *Zfp580* induced *Igf1* and *Igfbp3* mRNA (Figures 1D,F). Therefore, Zfp580 is a suppressor of *Igf1* and *Igfbp3* expression in endothelial cells.

### Increased Expression of Zfp580 Ameliorates Effects of OGD on Igf1 and Igfbp3 Expression

**FIGURE 2:**
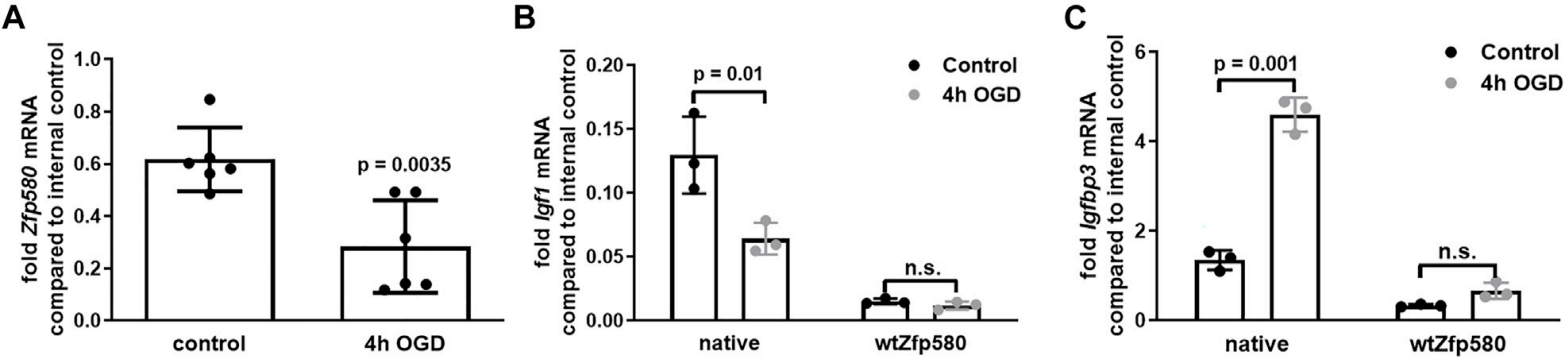
Zfp580 mediates *Igf1* and *Igfbp3* regulation after OGD. Endothelial bEnd.3 cells were subjected to 4 h of oxygen and glucose deprivation (OGD). Control cells were cultured under normal conditions in OGD buffer containing glucose. Expression levels of *Zfp580* **(A)**, *Igf1* **(B)** and *Igfbp3* **(C)** were determined by real-time RT-PCR immediately after OGD. **(A)**. *Zfp580* was suppressed by OGD. **(B,C)**. In unmodified cells, *Igf1* was suppressed and *Igfbp3* was induced by OGD. In a rescue experiment with ectopic increased expression of *Zfp580,* the effect of OGD on *Igf1* or *Igfbp3* expression was blunted. Means and scatter dot plots of independent experiments with standard deviation, two-tailed unpaired Students t-test **(A)**, or two-way ANOVA with Bonferroni multiple comparison test **(B,C)** are shown in the graphs.

To simulate a stroke *in vitro*, we exposed the cells to a 4-h period of combined oxygen and glucose deprivation (OGD). Cells cultured in OGD-buffer with glucose under normal conditions served as negative controls. Immediately after OGD, mRNA expression levels were determined by real-time RT-PCR. We identified a strong suppression of *Zfp580* mRNA after OGD (Figure 2A) and, consistently, *Igfbp3* mRNA was more than doubled (Figure 2C, left panel with native cells). However, *Igf1* was suppressed after OGD (Figure 2B, left panel with native cells). Next, we evaluated whether Zfp580 mediates the effects of OGD on *Igf1* and *Igfbp3* expression. Since *Zfp580* mRNA is strongly suppressed after OGD, we used bEnd.3 cells with exogenously increased expression of *Zfp580* in a rescue experiment to prevent the loss of Zfp580 after OGD. In cells cultured under control conditions, increased expression of Zfp580 suppressed *Igf1* and *Igfbp3* (Figures 2B,C). Increased *Zfp580* expression blunted the effect of OGD on *Igf1* and *Igfbp3* expression. Therefore, preventing Zfp580 loss after OGD interfered with Igf1 and Igfbp3 regulations after OGD.

### Genomic Ablation of Zfp580 has no Effect on Paracrine Cerebral *Igf1* and *Igfbp3* Expression and Endocrine Blood Levels *In Vivo*

**FIGURE 3:**
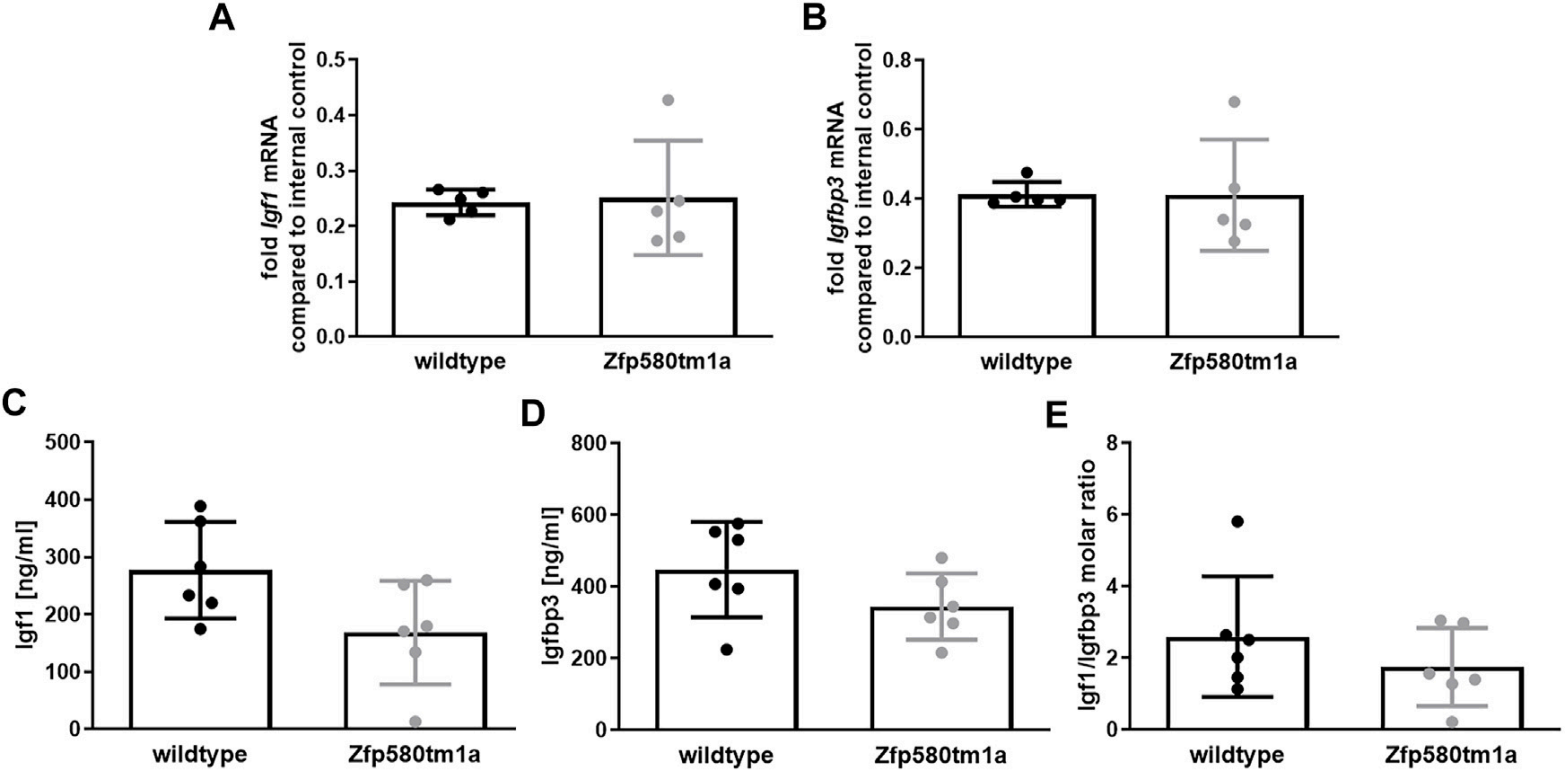
Zfp580 knockout has no effect on basal cerebral and systemic Igf1 and Igfbp3. In wildtype littermates and Zfp580 knockout mice, expression of *Igf1* and *Igfbp3* was determined from total brain lysate by real time RT-PCR **(A,B)**. Blood levels were determined by ELISA **(C,D)** and the molar ratio of Igf1: Igfbp3 calculated **(E)**. Knockout of Zfp580 had no effect on mRNA levels of *Igf1* and *Igfbp3* in the brain. There was no difference in the blood levels of Igf1 and Igfbp3 or the Igf1: Igfbp3 molar ratio. The graphs show the means and scatter dot plots of independent experiments with standard deviations, as well as the two-tailed unpaired Students t-test.

**FIGURE 4:**
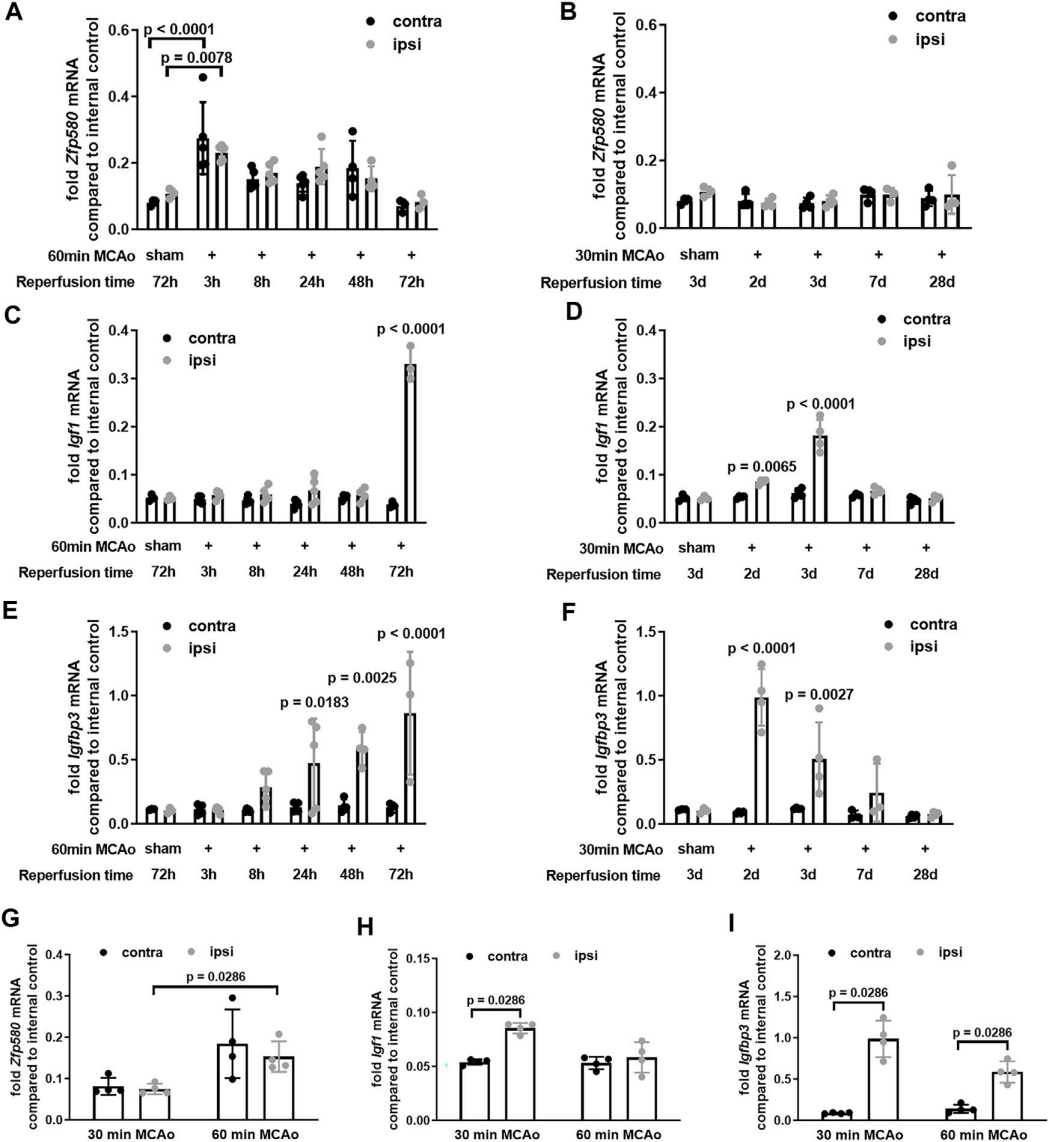
Igf1 and Igfbp3 are induced with delay in the ischemic hemisphere, whereas Zfp580 is globally induced throughout the brain early after stroke. Wildtype mice received MCAo for 30 min, or 60 min or a sham operation. Real-time RT-PCR was performed for *Zfp580* **(A,B,G)**, *Igf1* **(C,D,H)** and *Igfbp3* **(E,F,I)** from brain slices within the infarct area, separated into ipsi-lesional and contra-lesional hemispheres at the indicated reperfusion time-points. *Zfp580* is globally induced in both hemispheres early after 60 min MCAo up to 2 days after ischemia **(A)**. After 30 min MCAo, there was no significant regulation of Zfp580 **(B)**. *Igf1* rises selectively in the ischemic hemisphere starting after 2 d, with a maximum after 3 d and normalization after 7 d **(C,D)**. *Igfbp3* rises earlier, with a maximum after 2 d and normalization after 7 d **(E,F)**. At day 2, *Zfp580* induction was stronger after 60 min MCAo than after 30 min MCAo **(G)**, whereas there was only after 30 min MCAo an induction of *Igf1* in the ipsilesional hemisphere **(H)** and the induction of *Igfbp3* was less pronounced after 60 min MCAo compared to 30 min MCAo **(I)**. Means and scatter dot plots of independent experiments with standard deviation, two-way ANOVA with Dunnetts multiple comparison test **(A–F)**, or Mann-Whitney test **(G–I)** are shown in the graphs.

We determined the effects of Zfp580 genomic ablation on Igf1 and Igfbp3 *in vivo* using Zfp580 knockout mice (Zfp580tm1a^(EUCOMM)Wtsi^, a constitutive knockout mouse model from the EUCOMM project). Wild-type littermates served as controls. To assess paracrine effects, we extracted RNA from brain lysates and determined *Igf1* and *Igfbp3* mRNA levels by real-time RT-PCR. We identified no obvious differences between Zfp580tm1a and wild-type littermates in paracrine cerebral *Igf1* and *Igfbp3* expression (Figures 4A,B). Additionally, we determined blood levels of endocrine circulating Igf1 and Igfbp3 using ELISA. There was no difference in Igf1 and Igfbp3 blood levels or in the Igf1/Igfbp3 molar ratio between the genotypes (Figures 3C–E). Thus, under normal settings, genomic ablation of Zfp580 has no effect on paracrine or endocrine Igf1 or Igfbp3.

### Early After Stroke, Zfp580 is Globally Induced in the Brain, Whereas Igf1 and Igfbp3 Are Induced Late in the Ischemic Hemisphere

Next, we evaluated the effects of stroke on *Zfp580*, *Igf1 and Igfbp3* expression after 60 min of transient filamentous middle cerebral artery occlusion (MCAo) for short-term regulation up to 3 days in wild-type C56/BL6 mice. Additionally, we used a 30-min MCAo to evaluate long-term effects up to 28 days. Negative controls were sham operated animals that had the exact surgery process but were immediately removed of the filament. *Zfp580* expression was induced acutely after stroke, starting 3 h after ischemia (Figure 4A). Expression was induced globally in both hemispheres and normalized 3 days after ischemia. After 30 min of MCAO, there was no significant difference in *Zfp580* expression in the long-term (Figure 3B). *Igf1* expression was selectively induced in the ipsilesional hemisphere starting 2 days after ischemia (Figures 4C,D). Expression normalized between day three and day 7 after ischemia. Similarly, *Igfbp3* expression was selectively induced in the ischemic hemisphere, while induction occurred earlier starting 24 h after ischemia and persisted up to 7 days (Figures 4E,F). When we investigated the effects of MCAo duration on expression levels on day 2 following ischemia, we discovered that a 60-min MCAo induced Zfp580 to a greater extent than a 30-min MCAo (Figure 4G). *Igf1* was induced only after a 30 min MCAO (Figure 4H) at this early point of time. Induction of *Igfbp3* was less pronounced after a 60 min MCAO than after a 30 min MCAo (Figure 4I). Thus, early induction of *Zfp580* might inhibit *Igf1* and *Igfbp3* expression in the acute phase following stroke, but lowering *Zfp580* after 3 days may allow *Igf1* and *Igfbp3* expression through disinhibition. Moreover, higher levels of *Zfp580* might cause a reduced induction of *Igf1* and *Igfbp3* following a prolonged duration of MCAo.

### Zfp580 Differently Controls Paracrine Cerebral and Endocrine Circulating Igf1 and Igfbp3 Regulations After Stroke

**FIGURE 5:**
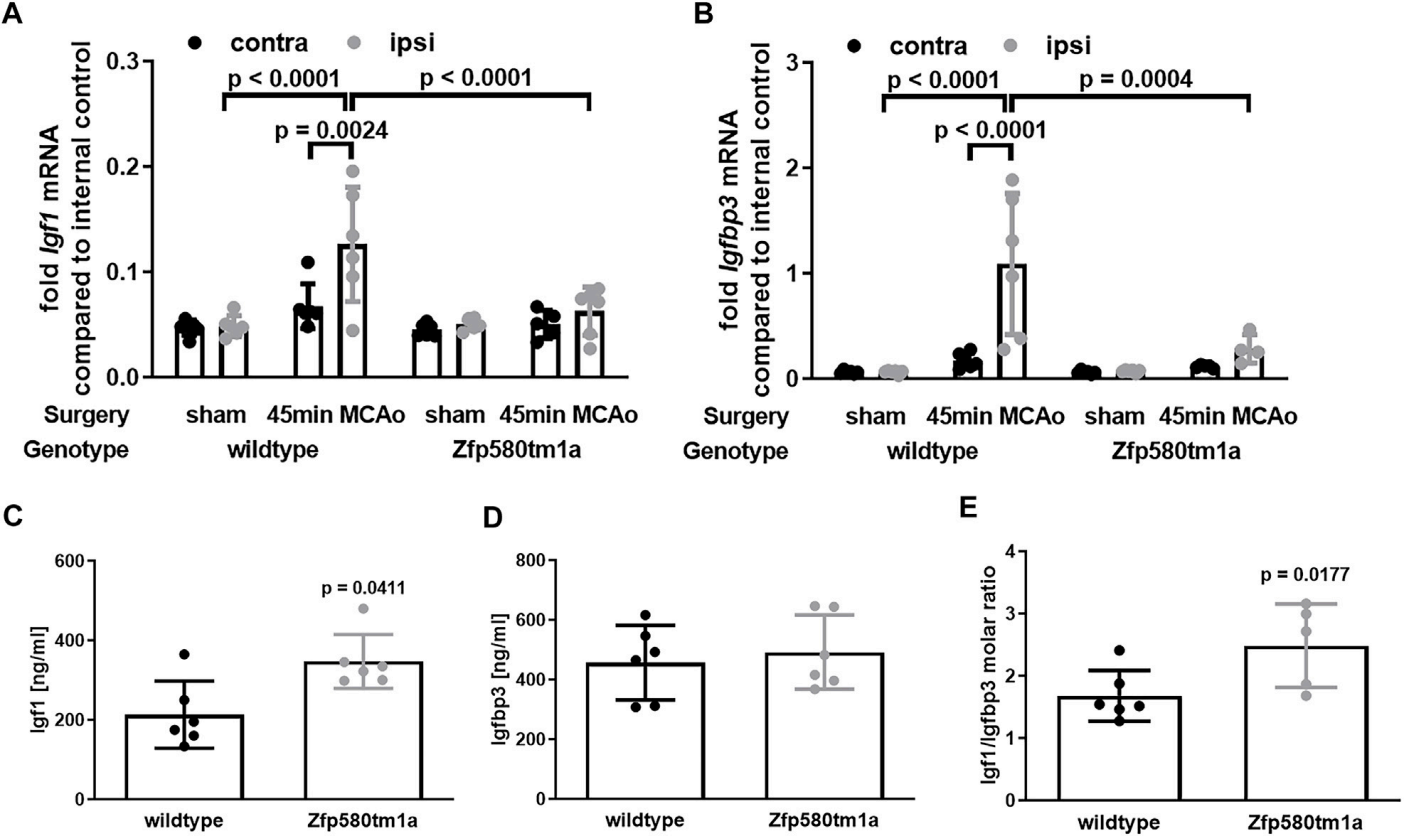
Zfp580 steers local cerebral and systemic regulation of Igf1 and Igfbp3 after stroke. Mice received a 45-min MCAo or sham operation. Analysis was done on day 2 after ischemia. Real-time RT-PCR was performed for *Igf1* **(A)** and *Igfbp3* **(B)** from brain slices within the infarct area, separated into ipsi-lesional and contra-lesional hemispheres from Zfp580 knockout mice and wildtype littermates. In wildtype mice, there is a strong induction of *Igf1* and *Igfbp3* expression in the ischemic hemisphere. Both were absent in the Zfp580tm1a mice. Systemic Igf1 and Igfbp3 protein were measured in blood samples by ELISA **(C,D),** and the molar ratio was calculated **(E)**. After MCAo, Igf1 blood levels were higher in Zfp580 knockout mice than in wildtype littermates. There was no difference for Igfbp3. The Igf1/Igfbp3 molar ratio was higher in Zfp580 knockout mice compared to wildtype littermates. Graphs show means and scatter dot plots of independent experiments with standard deviation, two-way ANOVA with Tuckey’s multiple comparison test **(A,B)**, Mann-Whitney test **(C)**, or two-tailed unpaired Student’s t-test **(D,E)**.

We determined the expression levels 2 days after 45 min MCAo in Zfp580 knockout mice to determine if Zfp580 has an effect on the paracrine cerebral Igf1 and Igfbp3 regulation following stroke. Wild-type littermates and sham-operated mice served as negative controls. In wild-type littermates, we again identified an induction of *Igf1* and *Igfbp3* in the ischemic hemisphere (Figures 5A,B). There was no observable induction of *Igf1* or *Igfbp3* in Zfp580 knockout mice (Figures 5A,B). We measured Igf1 and Igfp3 levels in the blood 2 days after ischemia using an ELISA to examine the effects of Zfp580 on post-ischemic endocrine regulation. After stroke, Igf1 blood levels were significantly increased in Zfp580 knockout mice (Figure 5C). However there was no difference in Igfbp3 blood levels between the two genotypes (Figure 5D). The Igf1/Igfbp3 molar ratio was greater in Zfp580 knockout mice than in wild-type littermates (Figure 5E). Thus, Zfp580 deletion resulted in reduced paracrine cerebral *Igf1* and *Igfbp3* expression, increased endocrine Igf1 blood levels, and consecutively increased Igf1/Igfbp3 molar ratios 2 days after stroke.

**FIGURE 6:**
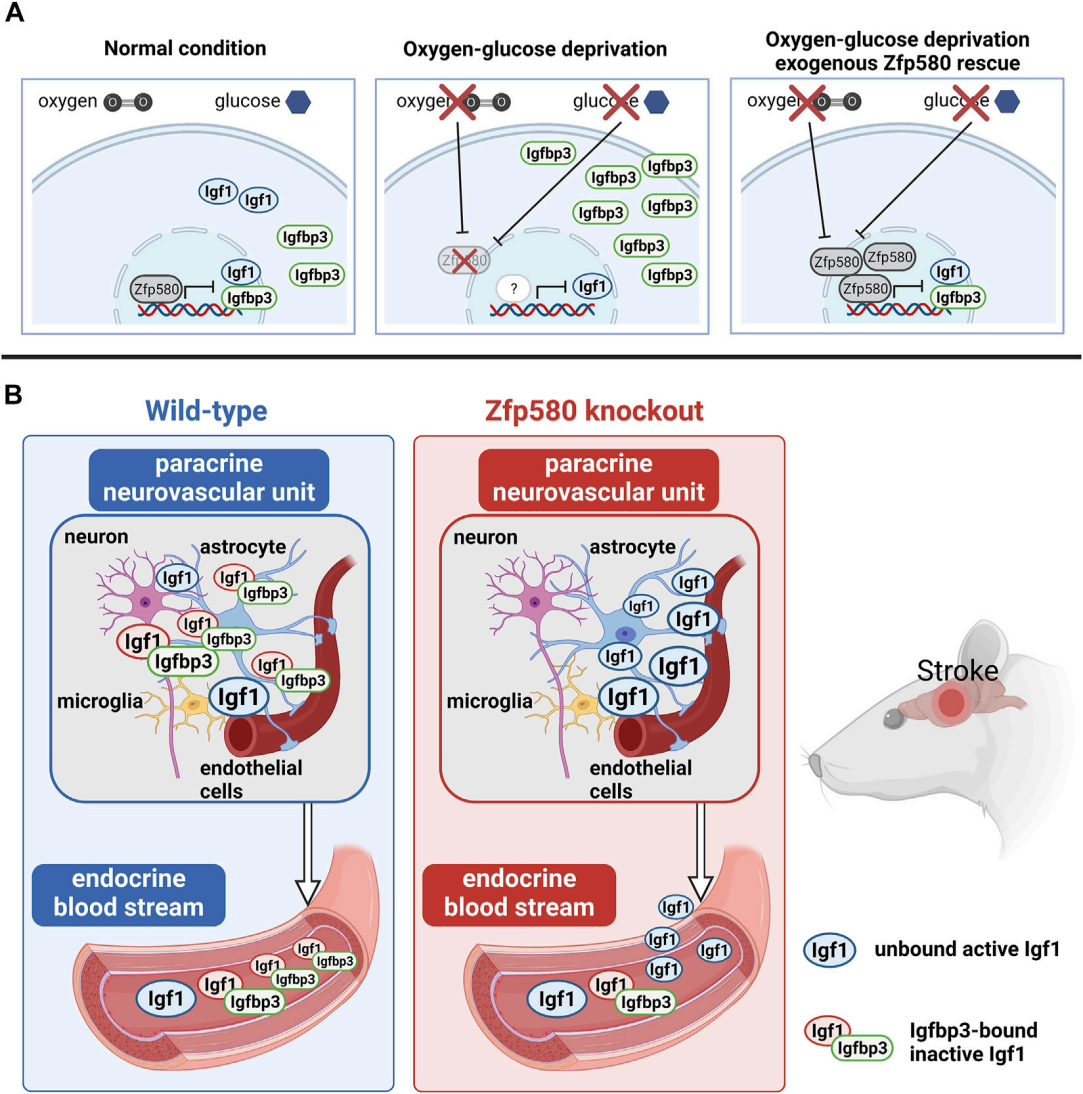
Illustration. **(A)** Under normal conditions, Zfp580 inhibited *Igf1* and *Igfbp3* expression. OGD suppressed *Zfp580* expression and dis-inhibited *Igfbp3*, whereas *Igf1* was suppressed. The OGD´s effect on *Igf1* and *Igfbp3* expression was blunted in a rescue experiment using exogenously increased *Zfp580* expression. **(B)** In wild-type mice, paracrine *Igf1* and *Igfbp3* were increased in the brain following stroke. The induction of paracrine *Igf1* and *Igfbp3* was absent in Zfp580 knockout mice, whereas endocrine Igf1 in the blood and the Igf1/Igfbp3 molar ratio were increased. As a result, Zfp580 knockout mice have an increased amount of endocrine unbound and active Igf1. Igf1 that is not bound to Igfbp3 can cross the blood-brain barrier, increasing cerebral Igf1.

## Discussion

We identified Zfp580 as a novel factor that differentially modulates paracrine and endocrine Igf1 and Igfbp3 responses after cerebral ischemia. Genomic Zfp580 deletion led to reduced paracrine cerebral Igf1 and Igfbp3, but increased endocrine circulating Igf1 and the Igf1/Igfbp3 molar ratio. We demonstrate in this study that Zfp580 interacts *via* Igf1 and Igfbp3 signaling with mechanisms relevant to stroke outcome.

*In vitro*, Zfp580 suppressed *Igf1* and *Igfbp3* expression in microvascular cerebral endothelial cells. Zfp580 is known to act on inflammatory and angiogenetic signaling pathways (Sun et al., 2010; Hoffmann et al., 2011; Luo et al., 2014; Stenzel et al., 2018). We identified growth hormone pathways as a new signaling pathway that is influenced by Zfp580. When modeling stroke *in vitro* by combined oxygen and glucose deprivation, *Zfp580* was suppressed in endothelial cells. Furthermore, *Igf1* expression was suppressed whereas *Igfbp3* expression was induced. When preventing suppression of Zfp580 by exogenously increasing expression of Zfp580, the effect of OGD on *Igf1* and *Igfbp3* expression was attenuated. This suggests that Zfp580 is involved in mediating the effects of OGD on *Igf1* and *Igfbp3* expression. However, additional research is required to identify the underlying mechanisms in greater detail. It is known that *Igfbp3* induces apoptosis and inhibits cell growth (Grimberg and Cohen, 2000), and that reduced *Igf1* leads to impaired cerebromicrovascular functions (Toth et al., 2015). Therefore, Zfp580 acts by Igf1 and Igfbp3 on mechanisms highly relevant for stroke outcome.

*In vivo*, Zfp580 genomic ablation had no effect on baseline paracrine, cerebral or endocrine circulating Igf1 and Igfbp3 levels in a constitutive Zfp580 knockout mouse. This might be explained by compensatory regulations. After stroke, Zfp580 was swiftly and universally induced in the brain, and normalized after 3 days in wild-type mice. In contrast, paracrine Igf1 and Igfbp3 were only induced in the ischemic hemisphere 3 days after ischemia. Since Zfp580 suppresses Igf1 and Igfbp3, early induction of Zfp580 might block the induction of Igf1 and Igfbp3 at earlier points in time. Normalization of Zfp580 after 3 days may dis-inhibit Igf1 and Igfbp3. Our finding that longer duration of MCAo boosted Zfp580 induction while lowering Igf1 and Igfbp3 induction supports this.

Prior to stroke, basal paracrine and endocrine Igf1 and Igfbp3 levels were unchanged, and endogenous regulation of Igf1 and Igfbp3 occurs later after stroke. As a result, genomic ablation of Zfp580 should not have any influence on the brain cytoprotection effects of Igf1 and Igfbp3 in the acute time window. However, after stroke, genomic ablation of Zfp580 led to a strongly reduced induction of paracrine *Igf1* and *Igfbp3* expression, whereas endocrine circulating Igf1 was increased. Endocrine circulating Igfbp3 in the blood remained unchanged. This causes an increased Igf1/Igfbp3 molar ratio, which results in more unbound and active Igf1 that can cross the blood-brain barrier. Igfbp3 induces apoptosis and inhibits cell growth (Grimberg and Cohen, 2000). Therewith a reduction of paracrine cerebral Igfbp3 as a result of genomic ablation of Zfp580 may be beneficial for stroke recovery. Additionally, reduced paracrine cerebral Igfbp3 increases the amount of active unbound Igf1 in the brain, which is known to improve outcome after stroke (Bondanelli et al., 2006; Aberg et al., 2011; Tang et al., 2014). However, paracrine cerebral Igf1 was reduced as well, which should worsen recovery after stroke. Albeit, circulating Igf1 can cross the blood-brain barrier. Increased levels of endocrine circulating Igf1 and the higher Igf1/Igfbp3 molar ratio may thus compensate for the decrease of paracrine cerebral Igf. This is supported by the fact that not only intracerebroventricular administration of Igf1 improves outcome after stroke, but even systemic injections of Igf1 are effective (Rizk et al., 2007; Fletcher et al., 2009; Chang et al., 2010). Additionally, Igf1 is important for proper neurovascular coupling (Toth et al., 2015). According to the recent STAIR recommendations (Savitz et al., 2019; Lyden et al., 2021), Zfp580 inhibition might be a target for post-thrombectomy cytoprotection aimed at both, neuronal survival and endothelial function, which are the primary components of the neurovascular unit. Furthermore, it may improve recovery and, as a result, functional long-term outcome.

The study’s primary limitation is that we present evidence using a constitutive knockout mouse model. Further research on the effects of Zfp580 on paracrine and endocrine Igf1 and Igfbp3 is required using cell-specific and inducible knockout mouse models. Additionally, we concentrated on the levels of paracrine cerebral Igf1 and Igfbp3 expression in this study. Additional post-translational effects on paracrine cerebral Igf1 and Igfbp3 may be evaluated in future research.

With this study, we identified Zfp580 as a novel modulator of Igf1 and Igfbp3, which differentially steers paracrine cerebral and endocrine systemic responses after stroke, leading to induced Igf1 signaling. Inhibition of Zfp580 might be a new treatment target to improve recovery after stroke.

## Data Availability

The original contributions presented in the study are included in the article, further inquiries can be directed to the corresponding authors.
